# Tigecycline-Induced Leukemoid Reaction in a Burn Patient: A Case Report

**DOI:** 10.7759/cureus.69689

**Published:** 2024-09-19

**Authors:** Bhushan R Patil, Sankalp Goel

**Affiliations:** 1 Plastic Surgery, Dr. D. Y. Patil Medical College, Hospital & Research Centre, Dr. D. Y. Patil Vidyapeeth (Deemed to be University), Pune, IND

**Keywords:** adverse drug reactions (adrs), drug-induced fever, leukemoid reaction, severe burn patient, tigecycline

## Abstract

In clinical practice, tetracycline antimicrobial agents often lead to adverse events with drug fever and leukemoid reaction (LR) being rare occurrences. Here, we present a case of tigecycline-induced LR in a burn patient, which we believe is the first reported case of tigecycline-induced drug fever and LR globally in a burn patient and second overall.

## Introduction

Tigecycline, an innovative tetracycline antimicrobial agent, is a derivative of minocycline with a 9-t-butylglycylamido modification. Tigecycline, featuring glycyl-amino substitutions at position 9, exhibits a broader spectrum of anti-infective activities [1]. In clinical practice, tetracycline antimicrobial agents often lead to adverse events, including gastrointestinal discomfort (nausea and vomiting), liver dysfunction, and renal impairment. However, drug fever and leukemoid reaction (LR) are rare occurrences associated with tetracycline antibiotics, with only a few reported cases linked to minocycline [2-5]. Here, we present a case of tigecycline-induced LR in a burn patient, which we believe is the first reported case of tigecycline-induced drug fever and LR globally in a burn patient and the second such case overall [6].

## Case presentation

**Figure 1:**
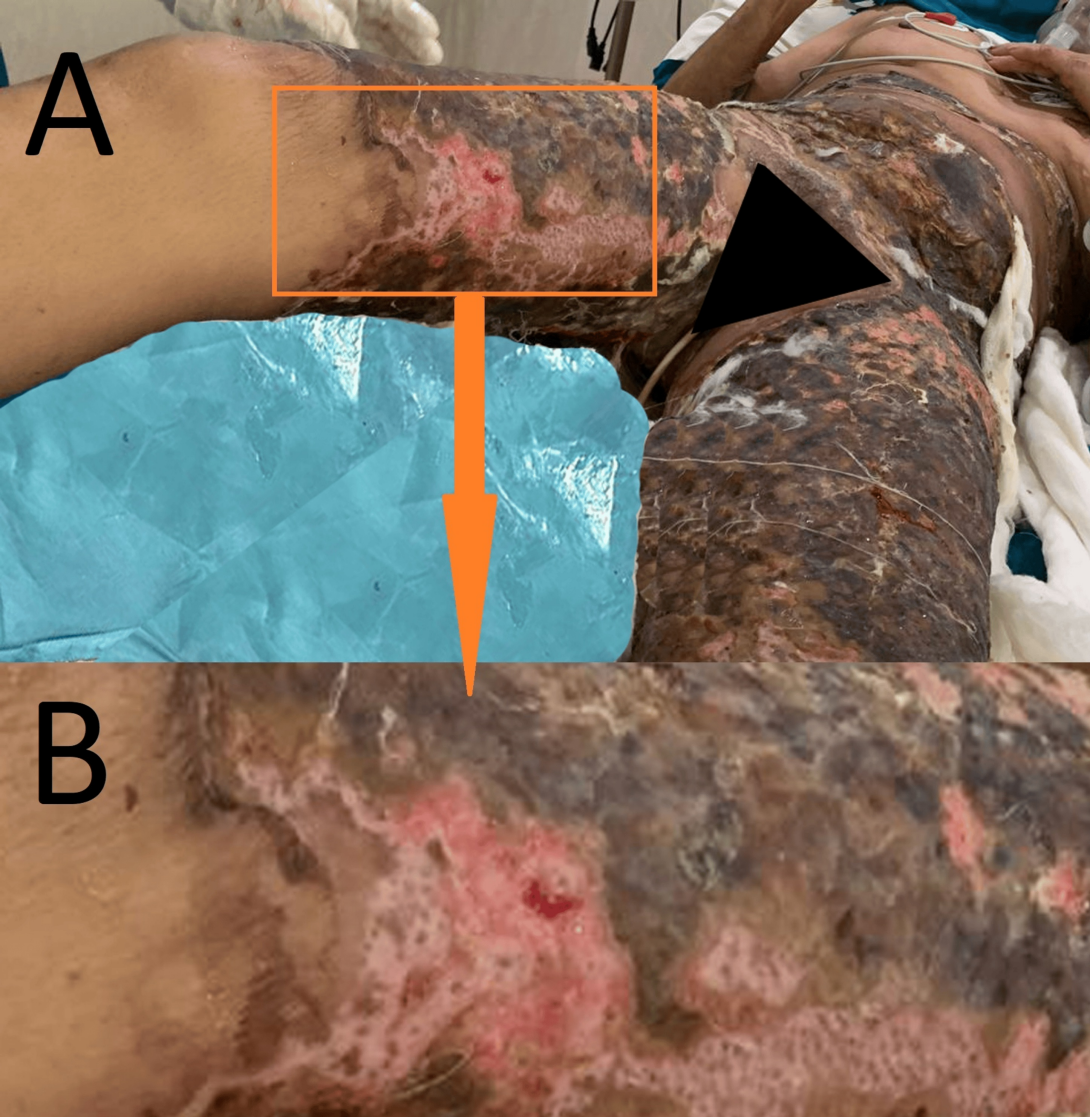
Clinical image of the patient with A) a whole clinical image of involved burnt areas with an orange box showing B) highlighted, zoomed in orange box area showing signs of wound healing and no subeschar collection Orange-colored box: highlighted and zoomed area to part B with orange-colored arrow

A female in her 50s presented with 30% second-degree deep flame burns over the abdomen, lower limbs, buttocks, and perineum. After admission to the Burns Intensive Care Unit (BICU), she was treated with fluid resuscitation and nanocrystalline silver-coated barrier dressing as per the protocol. Laboratory investigations were sent and monitored as per the protocol. The barrier dressing was adherent to the raw area. Prophylactic antibiotic coverage was given because of the large burn area and involvement of the perineum. Clinically, the patient was stable and the wounds appeared to be healing as evidenced by the peeling of the barrier dressings from the periphery of the wound. After 21 days, clinically, residual post-burn raw areas were assessed and prepared for coverage with a split-thickness skin graft (clinical image of the patient shown in Fig. 1). As per the protocol, regular wound cultures were taken and antibiotics were given as per their sensitivity. On post-burn day 24, the cultures showed methicillin-resistant staphylococcus aureus (MRSA), which was only sensitive to tigecycline. The patient was shifted from injection ceftazidime 1 g to injection tigecycline 100 mg stat, followed by 50 mg twice daily for 14 days.

**Figure 2:**
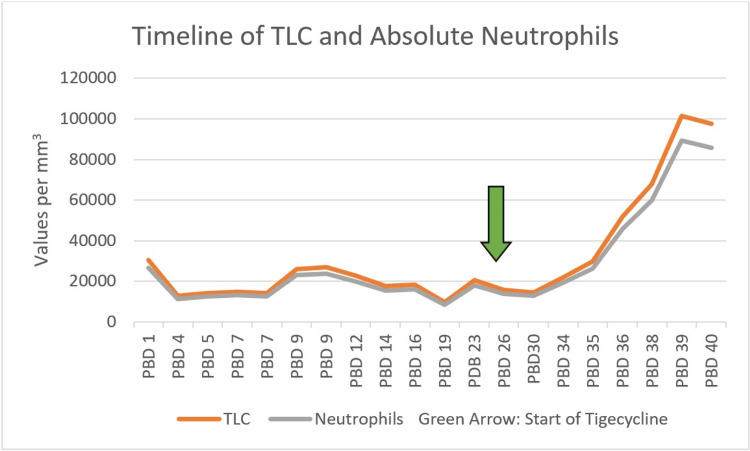
Timeline of the patient’s TLC and absolute neutrophil count throughout the hospital stay with emphasis on the response to inj. tigecycline PBD: post burn day, orange line: timeline of the total leukocyte count (TLC), gray line: timeline of neutrophils, green arrow: start of tigecycline (post burn day 26)

Within 10 days of starting tigecycline, the patient’s general condition remained satisfactory, but the patient started developing fever spikes. The patient’s total leucocyte count (TLC) surged from 14,600/µL to 21,800/µL with 90% neutrophil count. The patient’s TLC continued to rise and peaked at 101,500/µL over the next four days (the trend is shown in Fig. 2).

With the rise of TLC, there was suspicion of invasive infection, but the clinical picture was not congruent with the same. To aid in the diagnosis, another wound culture was taken and sent for culture. Leucocyte alkaline phosphatase (LAP) was also sent. The LAP score was 150, for which the normal range is 20-100, confirming an LR. The culture showed no growth. This further suggested that the leucocytosis was due to the LR to the drug tigecycline and not due to sepsis. Tigecycline was immediately discontinued, and the patient was started on appropriate corticosteroid treatment on PBD 39. Unfortunately, the patient succumbed to her burn wounds amidst the management of an LR on PBD 40.

## Discussion

An LR is a hematological condition characterized by an elevated white blood cell count exceeding 50,000 cells/μL. It occurs as a response to external factors rather than originating from the bone marrow [7,8]. LR is marked by a significant increase in mature neutrophils in the bloodstream and a notable shift to the left in the differential count [8]. The diagnosis of LR hinges on ruling out chronic myelogenous leukemia (CML) and chronic neutrophilic leukemia (CNL). Key distinguishing factors include the absence of basophilia, immature cells, elevated leukocyte alkaline phosphatase (LAP), and the absence of the bcr/abl translocation, which is commonly seen in CML. LRs primarily stem from severe infections, poisonings, malignancies, extensive bleeding, or acute hemolysis. LR can also occur in response to drug exposure (e.g., minocycline, recombinant hematopoietic growth factors, corticosteroids [9]) or exposure to toxins such as ethylene glycol [10].

After a thorough search through published literature, this case is the world's first case of a burn patient with tigecycline-induced LR. A review of the existing literature revealed numerous case reports of drug fever and LR linked to minocycline. It is suggested that minocycline acts as a cytokine that stimulates the movement of neutrophils from the bone marrow into circulation, leading to LR [2,5]. Minocycline-related case reports have indicated that systemic reactions can be severe, including high fever and LR, as well as generalized eruption, exfoliative dermatitis, and severe hepatitis, which may be fatal without prompt and appropriate intervention [5]. Given the structural similarities between tigecycline and minocycline, it is reasonable to postulate that they can induce similar hypersensitivity reactions.

Considering the patient's attributes, it is essential to include drug-induced hypersensitivity in the list of potential causes for fever and LR in the differential diagnosis. Older individuals, especially those administered multiple medications throughout their illness, face an elevated susceptibility to hypersensitivity reactions. Furthermore, tetracycline-induced hypersensitivity is linked to T-cell activation. Consequently, monitoring T-cell subsets can play a crucial role in promptly identifying hypersensitivity and expediting the diagnostic process. [5,11]. In our case, due to the patient succumbing to her burn wounds, we could not measure or monitor the T-cell subsets in time. If needed, discontinuing the presumed medication to validate the diagnosis remains a viable course of action. In situations involving severe and life-threatening adverse events, contemplating the addition of short-term, low-dose steroids to alleviate inflammation could be a viable consideration.

## Conclusions

In our case, the patient suffered from an LR after being subjected to treatment with tigecycline, which has never been reported before. This case demonstrates that burn patients treated with multiple antibiotics at different stages of treatment as per culture growth and sensitivity reports are vulnerable to developing a leukemoid reaction, especially to the tetracycline group of antibiotics. Abnormal spikes of fever and raised TLC incongruent with the clinical picture should prompt evaluation of a possible leukemoid reaction for prompt management.

## References

[1] Yaghoubi S, Zekiy AO, Krutova M (2022). Tigecycline antibacterial activity, clinical effectiveness, and mechanisms and epidemiology of resistance: narrative review. Eur J Clin Microbiol Infect Dis.

[2] Chatham WW, Ross DW (1983). Leukemoid blood reaction to tetracycline. South Med J.

[3] Kaufmann D, Pichler W, Beer JH (1994). Severe episode of high fever with rash, lymphadenopathy, neutropenia, and eosinophilia after minocycline therapy for acne. Arch Intern Med.

[4] Parneix-Spake A, Bastuji-Garin S, Lobut JB, Erner J, Guyet-Rousset P, Revuz J, Roujeau JC (1995). Minocycline as possible cause of severe and protracted hypersensitivity drug reaction. Arch Dermatol.

[5] MacNeil M, Haase DA, Tremaine R (199736). Fever, lymphadenopathy, eosinophilia, lymphocytosis, hepatitis, and dermatitis: a severe adverse reaction to minocycline. J Am Acad Dermatol.

[6] Shao QQ, Qin L, Ruan GR, Chen RX, Luan ZJ, Ma XJ (2015). Tigecycline-induced drug fever and leukemoid reaction: a case report. Medicine (Baltimore).

[7] Nimieri HS, Makoni SN, Madziwa FH, Nemiary DS (2003). Leukemoid reaction response to chemotherapy and radiotherapy in a patient with cervical carcinoma. Ann Hematol.

[8] Curnutte J, Coates T (2000). Disorders of phagocyte function and number.. Hematology, Basic Principles and Practice 3rd Ed.

[9] Ganti AK, Potti A, Mehdi S (2003). Uncommon syndromes and treatment manifestations of malignancy: case 2. Metastatic non-small-cell lung cancer presenting with leukocytosis. J Clin Oncol.

[10] Mycyk MB, Drendel A, Sigg T, Leikin JB (2002). Leukemoid response in ethylene glycol toxication. Vet Hum Toxicol.

[11] Guillon JM, Joly P, Autran B, Denis M, Akoun G, Debré P, Mayaud C (1992). Minocycline-induced cell-mediated hypersensitivity pneumonitis. Ann Intern Med.

